# Prognostic value of FDG-PET SUV changes in cervical cancer following radiation therapy: a retrospective cohort study

**DOI:** 10.1007/s00404-026-08330-5

**Published:** 2026-01-29

**Authors:** Claudia A. Bale, Janina V. Pearce, Xiaoyan Deng, Dipankar Bandyopadhyay, Nophar Yarden, Catherine Sport, Devin T. Miller, Leslie M. Randall, Emma Fields, Stephanie A. Sullivan

**Affiliations:** Virginia Commonwealth University Medical Center, 1250 E Marshall St, Richmond, VA 23219 USA

**Keywords:** SUV change, Cancer recurrence, Lymph node avidity, Metabolic imaging, Standardized uptake value

## Abstract

**Purpose:**

This study sought to determine the relationship between cervical cancer recurrence and post-treatment change in standardized uptake value (SUV) of 18F-2-fluoro-2-deoxy-D-glucose-positron emission tomography (FDG-PET) in the cervix and lymph nodes.

**Methods:**

This retrospective cohort study included patients who received curative intent radiation therapy for biopsy-proven stage I–IVA locally advanced cervical cancer at a single tertiary referral center from 2009 to 2021. The exposure was percent change in SUV from pre- to post-treatment FDG-PET scans at the cervix and lymph nodes. The primary outcome was recurrence rate, and secondary outcomes were overall and progression-free survival. Firth’s penalized logistic regression and Cox proportional hazards models were used to assess associations.

**Results:**

55 patients met eligibility criteria. Recurrence rate was 27% (15/55); of these, 33% had local recurrence (5/55) and 67% had distant recurrence (10/55). Median percent decrease of cervical SUV after treatment in those with and without recurrence was similar (71.4 vs 68.8, *p* = 0.89); this remained consistent when analyzing those with local recurrence only (70.5, *p* = 0.95). When the percent decrease in cervical SUV was examined in intervals (< 25%, 25–50%, 50–75%, > 75%), this was also not predictive of local (*p* = 0.91) or overall (*p* = 0.75) recurrence. Median percent decrease at the most avid and distant lymph node in those with and without recurrence was not significantly different (*p* > 0.05). Neither change in cervical nor lymph node SUV was associated with overall or progression-free survival.

**Conclusion:**

Changes in SUV after treatment may not be a reliable stand-alone marker for predicting recurrence or survival in locally advanced cervical cancer after treatment with radiation therapy.

## Introduction

Cervical cancer is the 4th most frequently diagnosed cancer and cause of cancer death in women worldwide [1, 2]. In 2018, the International Federation of Gynecology and Obstetrics (FIGO) updated its guidelines, highlighting the importance of imaging as part of clinical staging and planning optimal treatment for patients with cervical cancer [3]. Positron emission tomography with radiotracer uptake of 18F-2-fluoro-2-deoxy-D-glucose (FDG-PET) is a tool for staging primary and recurrent cervical cancer that detects physiologic changes based on the degree of tumor uptake and metabolism of glucose [4].

Of patients who present with cervical cancer, an estimated median 37% present with locally advanced disease requiring primary radiation therapy with or without chemotherapy [5] for curative intent treatment. Patients now routinely obtain FDG-PET pre- and post-treatment to assess response; however, there are inconsistent data on how to interpret residual avidity in the post-treatment period, leaving providers with a clinical conundrum and patients at risk for false-positive or false-negative interpretations [6].

Post-treatment FDG-PET avidity in the cervix has been found to be a significant independent predictor of survival for patients with cervical cancer [7–9] with prognostic value in assessing patterns of treatment failure and eligibility for salvage therapy [9]. Optimal timing of post-treatment imaging is still undefined and has varied in other studies with combined FDG-PET/computed tomography (CT) scanning [10–13]. One retrospective study found that post-treatment FDG-PET avidity alone was the most significant prognostic factor for developing metastatic disease and survival outcomes but with large variation in time from treatment to follow-up (range 1 to 12 months, mean 3 months) [7]. In a prospective study, Schwarz et al. demonstrated that 3-month post-treatment FDG-PET avidity was a significant predictor of survival [14]. While these studies highlight the importance of the presence or lack of avidity in post-treatment scans, only one study [15] quantified change in maximum SUV of FDG in the cervix as a percentage of residual activity and found no significant difference between a cohort of 20 patients with locally advanced cervical cancer (8 of whom developed recurrence). No studies have attempted to quantify percent changes in SUV in the cervix and lymph nodes in the context of recurrence or survival outcomes.

The objective of this study was to determine the relationship between cancer recurrence and the post-treatment percent change in SUV of FDG-PET in both the cervix and the lymph nodes.

## Methods

### Patient selection

Following public registration of the investigation (researchregistry10267) and institutional review board approval (IRB HM20024055), a retrospective cohort study was performed at a single-site urban, safety net, tertiary referral institution with National Cancer Institute (NCI) designation. Patients at least 18 years of age with biopsy-proven stage I–IVA locally advanced cervical cancer between 2009 and 2021 who received curative intent radiation therapy at our tertiary care academic institution were eligible for inclusion. Additional inclusion criteria required patients to have both a pre-treatment scan (before starting radiation therapy) and at least one post-treatment FDG-PET scan completed 2–5 months after their radiation therapy end date. Cervical cancer was classified according to the 2018 FIGO staging system. Patients with metastatic disease at diagnosis, a history of hysterectomy, or who received palliative intent treatment were excluded.

All patients included in the final analysis (55/55, 100%) completed the prescribed radiation therapy regimen and underwent both pre-treatment and post-treatment FDG-PET imaging as required by the study protocol. There was no loss to follow-up for the primary outcome assessment of post-treatment imaging changes. For survival analysis, clinical follow-up duration varied based on treatment dates within our study period (2009–2021), with complete follow-up data available for all patients through their last clinical visit or date of death.

### Clinical data

Clinical data collected included age and body mass index (BMI) at diagnosis, race, ethnicity, marital status, employment status, urban (population > 2500) vs rural (< 2500) home address, medical comorbidities (diabetes, HTN, HIV, sarcoidosis, tobacco use), receipt of chemotherapy and/or immunotherapy, tumor size and histology, and cancer stage [16–18].

Local recurrence was defined as regrowth of tumor within the pelvis, including the cervix, vaginal vault, paravaginal area, or parametria, and excluded regional nodal recurrence and distant metastases.

Overall survival and progression-free survival were measured from the date of radiation therapy initiation to the date of last clinical follow-up or date of death for overall survival, or date of recurrence for progression-free survival. Recurrence was confirmed histologically and/or radiologically.

The same PET scanner was used from 2013 to the present; however, prior to 2013, a different scanner was in use. Of the 55 patients included in this study, 7 completed their pre and post-treatment scans prior to 2013. SUV measurements were obtained by radiologists on staff during the study period, with no changes in protocol for assessing SUV values with the change in scanner. Radiologists reviewed imaging as routine in their clinical practice in a standardized manner without direct involvement in how reported SUV values would be used to guide clinical decision-making.

All patients were treated with definitive intent with external beam radiotherapy. Patients either received 3D or intensity-modulated radiation therapy (IMRT) and either intracavitary or hybrid intracavitary/interstitial brachytherapy. Prior to June 2019, radiation therapy doses were prescribed using the traditional “point A” method with a biologically equivalent dose in 2 Gy fractions (EQD2) of 85 Gy to the tumor; 34 patients (62%) were treated with this approach. After June 2019, the practice transitioned to volumetric (3D image-guided) prescriptions, targeting an EQD2 of 85 Gy to the tumor, with PET-avid lymph nodes boosted to 54–59.4 Gy; 21 patients (38%) were treated with this modern approach.

Approximately 60 min (accepted range 55–75 min) after intravenous administration of 10–15 mCi of F-18 fluorodeoxyglucose, three-dimensional emission acquisition of the body extending from the skull base to mid-thighs was obtained. Blood glucose was measured prior to tracer injection. Noncontrast, low-dose CT acquisition was obtained for attenuation correction of the emission acquisition and anatomical localization of tracer distribution. A maximum intensity projection reconstruction of the PET acquisition was made for display as a rotating whole-body volumetric image. Tomographic images were computer generated and displayed from transaxial, coronal, and sagittal planes. PET and CT images were fused to aid in determining the anatomical location of F-18 FDG tracer distribution. To quantitate abnormal FDG uptake, F-18 was measured in the region of abnormality, normalized to the administered dose and the patient’s body weight, and expressed as a SUV.

The SUVs of avid lymph nodes were reviewed in the radiology imaging reports with the most avid lymph node selected based on the highest reported SUV, and the most distant lymph node was selected based on the most anatomically distant lymph node from the cervix. Pre- and post-treatment FDG-PET scans were assessed by radiology providers, and percent differences in the SUV at the cervix, the most avid lymph node, and the most distant lymph node were calculated and used as the independent variables for analyses.

### Statistical analysis

This study was conducted and reported following the Strengthening the Reporting of Observational Studies in Epidemiology (STROBE) guidelines for cohort studies [19]. Due to a non-normal distribution, the Wilcoxon non-parametric test was implemented for comparison of SUV distribution-related disease recurrence. Patient demographics and tumor characteristics between those with and without recurrence were compared using either non-parametric median two-sample tests, Cochran–Mantel–Haenszel tests, Fischer’s exact tests, or Chi-squared tests as appropriate.

To determine the effect of percent decrease of cervical SUV in combination with other covariates on recurrence risk, logistic regressions were performed, for the outcomes of overall recurrence as well as local recurrence only. The main independent variable was the percent decrease of cervical SUV, and the 17 aforementioned covariates were considered. Given the low number of recurrence events (*n* = 15 total; 5 local, 10 distant) relative to the number of covariates, Firth’s penalized likelihood method was used for logistic regression analyses to reduce small-sample bias and address potential separation issues. Models that did not converge with covariates were re-run as univariate Firth models. Tumor size and FIGO stage were included as covariates in multivariable models based on their established prognostic significance. The same analysis was conducted with percent decrease in SUV of the most distant node and again for the most avid node. Percent change in cervical SUV was additionally analyzed in discrete intervals (< 25%, 25–50%, 50–75%, > 75%) using the Cochran–Mantel–Haenszel test method.

The survival outcomes were estimated using the Kaplan–Meier method. The Cox proportional hazards model was conducted separately for the overall survival and the progression-free survival analyses. Multivariate analyses of the covariate factors were performed with results expressed as hazard ratios (HR) with 95% confidence intervals (CI) with a two-sided significance level of 5% (*p* < 0.05). All analyses were conducted using SAS (Statistical Analysis System, version 9.4) software.

## Results

### Patient characteristics

Median patient age at the time of treatment was 46 years (range 22–83) and median BMI was 29.3 kg/m² (range 17.7–50.8). Histology included 87% squamous cell carcinoma (48/55), 11% adenocarcinoma (6/55), and 2% adenosquamous carcinoma (1/55). Post-treatment FDG-PET scans were performed between 2 and 5 months (mean 3.5 months) after treatment completion for all patients. As this was a retrospective imaging analysis study, no study-specific interventions beyond standard clinical care were performed. Therefore, no study-related adverse events occurred. Standard treatment-related toxicities from radiation therapy and concurrent chemotherapy were managed according to institutional protocols but were not specifically analyzed as part of this imaging-focused investigation. No complications were reported during the FDG-PET imaging procedures themselves for any of the 55 patients included in the analysis. Overall recurrence rate was 27% (15/55); of these, 33% had local recurrence (5/55) and 67% had distant recurrence (10/55). All but 2 patients received chemotherapy, and 12 patients (7 disease free, 5 recurrence) received immunotherapy with radiation therapy and chemotherapy on clinical trial. All characteristics are presented in Table 1.

**Table 1:** Patient demographics and tumor characteristics. P values: Wilcoxon rank-sum tests (continuous variables), Fisher’s exact/Chi-squared tests (categorical variables)

*Patient characteristics*	Patients (n, %)			
	Total (*n* = 55)	Without disease recurrence (*n* = 40)	With disease recurrence (*n* = 15)	*P* value
Age at diagnosis, years				0.40
Median	46	47	42	
Range	22–83	22–83	27–76	
Race				0.59
Caucasian	27	18 (66.67%)	9 (33.33%)	
African American	22	17 (77.27%)	5 (22.73%)	
Other	6	5 (83.33%)	1 (16.67%)	
Ethnicity				1.00
Hispanic	1	1 (100%)	0 (0%)	
Non-Hispanic	54	39 (72.22%)	15 (27.78%)	
Clinical stage				0.012
I	7	7 (100%)	0 (0%)	
II	28	22 (78.57%)	6 (21.43%)	
III	15	6 (40%)	9 (60%)	
IVA	1	1 (100%)	0 (0%)	
Unknown	4	4 (100%)	0 (0%)	
Histology				0.79
Squamous	48	35 (72.92%)	13 (27.08%)	
Adenosquamous	6	4 (66.67%)	2 (33.33%)	
Adenocarcinoma	1	1 (100%)	0 (0%)	
Tumor grade				0.47
1	5	4 (80%)	1 (20%)	
2	15	9 (60%)	6 (40%)	
3	14	12 (85.71%)	2 (14.29%)	
Unknown	21	15 (71.43%)	6 (28.57%)	
Tumor size, mm				0.74
Median	58	57	58	
Range	20–100	20–100	40–90	
Pre-treatment Lymph node involvement				0.36
Yes	35	24 (68.57%)	11 (31.43%)	
No	21	16 (80%)	4 (20%)	
Chemotherapy regimen				0.47
Cisplatin	53	39 (73.58%)	14 (26.42%)	
None	2	1 (50%)	1 (50%)	

### Pre- and Post-treatment FDG uptake and recurrence

The comparison of the percent changes in pre- and post-treatment cervical FDG uptake, most distant, and most avid lymph node as predictors of recurrence are shown in Table 2.

**Table 2:** Percent changes in the cervical, most distant, and most avid lymph node max SUV in patients with and without disease recurrence. P values for continuous variables calculated using Wilcoxon rank-sum tests due to non-normal distribution. P values for interval analyses calculated using Cochran–Mantel–Haenszel tests. See Table 3 for Firth’s penalized logistic regression results

*Max SUV changes*	Without disease recurrence (*n* = 40)	With disease recurrence (*n* = 15)	P value
Cervix continuous interval			0.887 0.753
N for analysis	40	15	
Mean, pre-treatment SUV	17.66	16.13	
Mean, post-treatment SUV	4.05	6.99	
Mean, % change	65.86	54.66	
Median, % change	71.36	68.82	
Standard deviation	29.95	67.14	
Most distant lymph node			0.673
N for analysis	23	9	
Mean, pre-treatment SUV	5.15	5.57	
Mean, post-treatment SUV	0.63	1.15	
Mean, % change	82.44	80.00	
Median, % change	100	100	
Standard deviation	35.48	31.28	
Most avid lymph node			0.274
N for analysis	23	11	
Mean, pre-treatment SUV	5.69	7.96	
Mean, post-treatment SUV	0.98	2.45	
Mean, % change	79.69	70.65	
Median, % change	100	63.68	
Standard deviation	33.83	30.81	

Median percent decrease of cervical SUV after treatment in those with and without recurrence was similar (71.4 vs 68.8, *p* = 0.887); this remained consistent when analyzing those with local recurrence only (70.5 vs 68.8, *p* = 0.953). Using Firth’s penalized logistic regression with tumor size and stage as covariates, percent change in cervical SUV was not a significant predictor of overall recurrence (OR: 0.992, 95% CI: 0.977–1.008, *p* = 0.355). The multivariable model for local recurrence did not converge due to the small number of events (*n* = 5); in univariate Firth analysis, percent change in cervical SUV was not significantly associated with local recurrence (OR: 0.997, 95% CI: 0.980–1.014, *p* = 0.744). Multivariable models for local recurrence with lymph node predictors did not converge. Neither tumor size nor stage was significant predictors in any model (all *p* > 0.05).

When the percent decrease in cervical SUV was examined in intervals (< 25%, 25–50%, 50–75%, > 75%), the Cochran–Mantel–Haenszel test showed that the overall recurrence rate was not significantly different among the intervals (*p* = 0.753); this remained consistent when analyzing those with local recurrence only (*p* = 0.91). The multivariate logistic regression results showed that the interval percent decrease in cervical SUV was not a significant indicator for both overall and local recurrence (*p* = 0.7357 and 0.9852, respectively). No covariates were significant predictors of recurrence (*p* > 0.05 for all).

For the lymph nodes, the median percent decrease of SUV at the most avid and most distant lymph node from pre- to post-treatment PET in those with and without recurrence was not significantly different (*p* > 0.05 for both) for the 34/55 patients with nodal involvement. Using Firth’s penalized logistic regression with tumor size and stage as covariates, the percent change of SUV in the most distant node was not a significant predictor of distant recurrence (OR: 0.995, 95% CI: 0.972–1.019, *p* = 0.681) or overall recurrence (OR: 0.997, 95% CI: 0.974–1.020, *p* = 0.795). Similarly, the percent change of SUV at the most avid lymph node was not a significant predictor of distant recurrence (OR: 0.987, 95% CI: 0.963–1.011, *p* = 0.288) or overall recurrence (OR: 0.993, 95% CI: 0.970–1.017, *p* = 0.560). Multivariable models for local recurrence with lymph node predictors did not converge due to the small number of local recurrence events (*n* = 5); univariate Firth analyses also showed no significant associations (all *p* > 0.05). Neither tumor size nor stage was a significant predictor in any converged model (Table 3).

**Table 3:** Firth’s penalized logistic regression results for SUV percent change as predictor of recurrence. Models adjusted for tumor size (mm) and FIGO stage. Models for local recurrence with covariates did not converge due to small event counts (*n* = 5); univariate Firth results shown

Predictor	Outcome	OR	95% CI	*P *value
Cervix SUV % change	Overall recurrence	0.992	0.977–1.008	0.355
Cervix SUV % change*	Local recurrence	0.997	0.980–1.014	0.744
Distant LN % change	Distant recurrence	0.995	0.972–1.019	0.681
Distant LN % change	Overall recurrence	0.997	0.974–1.020	0.795
Distant LN % change*	Local recurrence	1.005	0.964–1.047	0.824
Avid LN % change	Distant recurrence	0.987	0.963–1.011	0.288
Avid LN % change	Overall recurrence	0.993	0.970–1.017	0.560
Avid LN % change*	Local recurrence	1.012	0.975–1.050	0.526

^*^Univariate Firth model (multivariable model with covariates did not converge). LN = lymph node; OR = odds ratio; CI = confidence interval

### Pre- and Post-treatment FDG uptake and survival

**Fig. 1:**
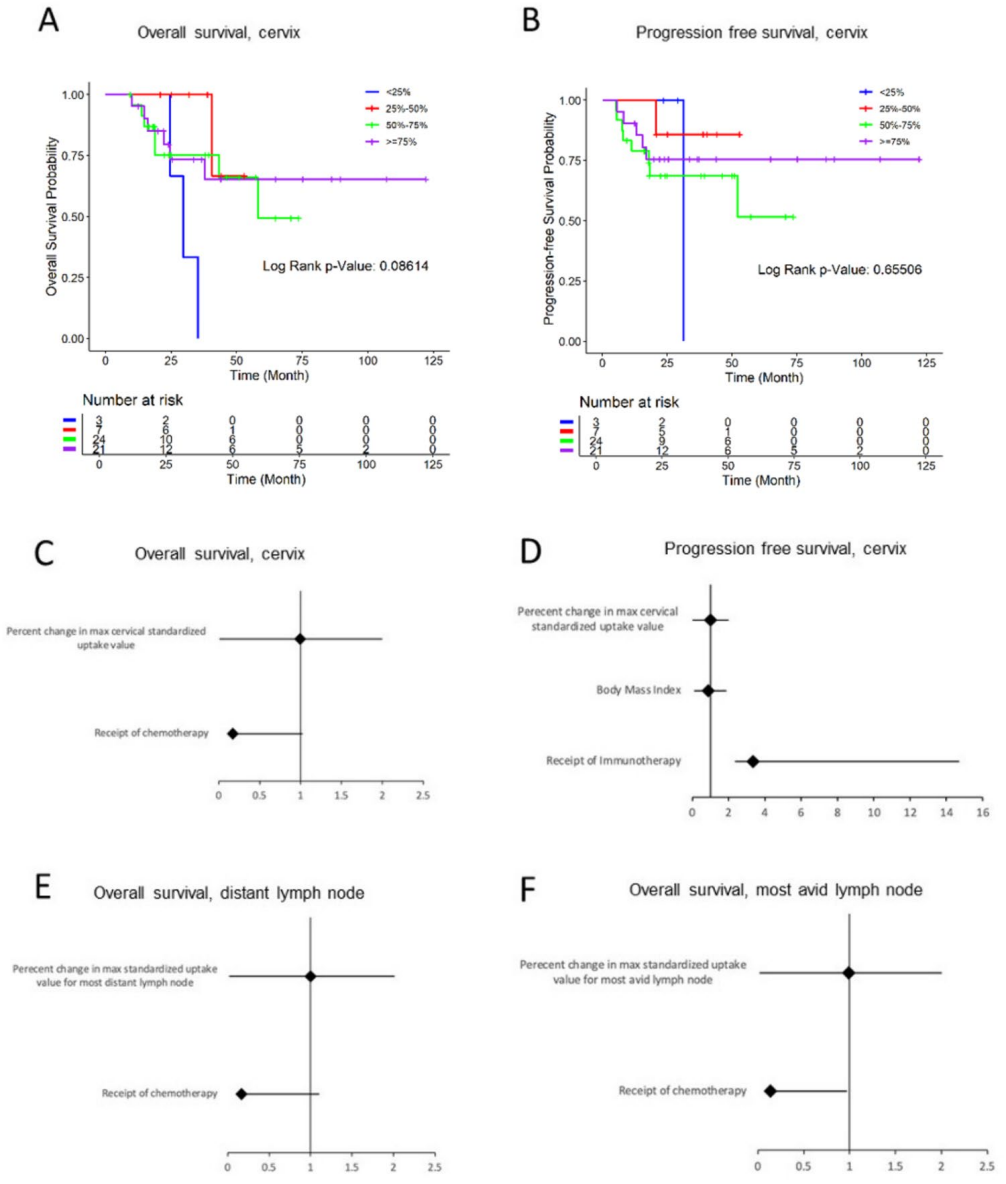
Kaplan–Meier curves based on categorization of percent change of cervical uptake values for **A** overall survival, **B** progression-free survival. Forest plots demonstrating hazard ratios for covariates related to **C** cervical uptake changes and overall survival, **D** cervical uptake changes and progression-free survival, **E** distant lymph node uptake changes and overall survival, **F** most avid lymph node uptake changes and overall survival. Kaplan–Meier curves (**A**, **B**) with log-rank tests and forest plots (**C**–**F**) showing hazard ratios from Cox models

Patients with and without recurrence differed in overall survival (*p* < 0.0001) and progression-free survival (*p* < 0.0001) outcomes as expected. However, the interval percent decrease in cervical SUV was not significantly associated with overall survival (Fig. 1A) or progression-free survival (Fig. 1B). In addition, percent changes in pre- and post-treatment cervical FDG uptake, most distant, and most avid lymph node as continuous variables were not associated with overall survival or progression-free survival (Table 4, with visual representation in Fig. 1C–F).

**Table 4:** Percent changes in max standardized uptake value and survival outcomes. Hazard ratios from Cox proportional hazards models

*Max SUV changes*	Overall survival		Progression-free survival	
	Overall survival hazard ratio HR (95% CI)	*P* value	Progression-free survival hazard ratio HR (95% CI)	*P* value
Cervix	0.994 (0.987, 1.001)	0.1199	0.997 (0.988, 1.006)	0.5361
Most distant lymph node	0.997 (0.983, 1.012)	0.7288	0.999 (0.981, 1.018)	0.9319
Most avid lymph node	0.994 (.980, 1.008)	0.3868	0.994 (0.978, 1.010)	0.4572

In multivariate analysis of percent change of cervical SUV, receipt of chemotherapy was the only covariate that was a significant predictor of longer overall survival (HR 0.17, *p* = 0.031), while higher BMI was the only covariate that was a significant predictor of longer progression-free survival (HR 0.89, *p* = 0.02). Receipt of immunotherapy was associated with shorter progression-free survival but was not statistically significant (HR 3.348, *p* = 0.053). Examining percent change in cervical SUV in intervals, no covariates were significant for overall survival, while again a higher BMI was significantly associated with longer progression-free survival (HR 0.90, *p* = 0.034) and receipt of immunotherapy was significantly associated with shorter progression-free survival (HR 3.70, *p* = 0.042) (Table 5).

**Table 5:** Significant predictors of overall or progression-free survival. Hazard ratios from multivariate Cox models with stepwise selection

*Overall survival*	Overall survival hazard ratio HR (95% confidence interval)	*P* value
Cervix, continuous chemotherapy	0.172 (0.035, 0.853)	0.0312
Most distant lymph node chemotherapy	0.165 (0.029, 0.939)	0.042
Most avid lymph node Chemotherapy	0.139 (0.023, 0.834)	0.031
*Progression-free survival*	Progression-free survival hazard ratio HR (95% confidence interval)	P value
Cervix, continuous BMI	0.891 (0.808, 0.982)	0.0198
Cervix, interval immunotherapy BMI	3.689 (1.051, 12.945) 0.901 (0.818, 0.992)	0.0416 0.0337

In multivariate analysis of percent change of lymph node SUV, of the covariate data collected, chemotherapy was a significant predictor of longer overall survival for the percent change of SUV for most distant node (HR 0.17, *p* = 0.042) and most avid node (HR 0.14, *p* = 0.031) (Table 4). No other covariates were significant predictors for overall survival nor progression-free survival.

## Discussion

### Summary of main results

Percent change of SUV in the cervix, the most avid lymph node, and the most distant node were not significant predictors of overall or local recurrence compared to those without disease recurrence, nor were they individual predictors of overall or progression-free survival in this cohort. In multivariate analysis examining percent change of cervical max SUV, receipt of chemotherapy and higher BMI were significant predictors of longer overall survival and longer progression-free survival, respectively. When examining interval percent change of cervical max SUV, higher BMI was again a significant predictor of progression-free survival, and receipt of immunotherapy was a significant predictor of shorter progression-free survival. For percent change in SUV for the most distant and most avid lymph node SUV, receipt of chemotherapy was a significant predictor for longer overall survival.

### Results in the context of published literature

FDG-PET has become a tool to detect cervical cancer recurrence by assessing SUV to monitor metabolic response to irradiated areas and assess for new or progressive disease [7]. A 2013 meta-analysis by Zhao and colleagues [21] concluded that SUV has predictive value in survival outcomes for guiding treatments; however, individual studies were small with heterogeneous samples that yielded conflicting results. Twelve of these studies examined max SUV of pre-treatment scans, while two studies used max SUV of post-treatment scans as prognostic factors of disease recurrence and survival outcomes. Only one study [15] collected SUVs from both pre- and post-treatment scans and found no significant differences between the percent residual activity of cervical max SUV in those with or without recurrence in a cohort of 20 patients. This study was the closest to our study design with similar results. In another meta-analysis by Sarker et al. [22] (with 6 of 16 studies overlapping with Zhao’s meta-analysis [21]), cervical max SUV was not a significant independent prognostic factor in the multivariate analyses conducted, except in 2 of 14 studies. They concluded that high SUV (≥ 13.4) should be considered at increased risk for recurrence; therefore, there is no overall clinical consensus within the field to determine what change in max cervical SUV confirms persistent or recurrent disease.

We chose to investigate the percent change of cervical max SUV between pre-treatment and post-treatment scans as a continuous variable and in discrete intervals of percentage change rather than focusing on a singular cut-off value on pre- or post-treatment scans. Although percent change of SUV between pre- and post-treatment scans was not a predictor of recurrence in our cohort, given our modest sample size and variability in SUV change among our patient population, it is difficult to draw conclusions about the prognostic utility of percent change in SUV.

Limited literature exists examining lymph node SUV max as a predictor of cervical cancer recurrence. Onal et al. [23] demonstrated that higher pelvic lymph node SUV was significantly associated with a higher risk of disease recurrence and worse survival. Two studies focused on cervical cancer patients with pelvic lymph node metastasis [24, 25]. Yen et al. [24] noted that SUV max > 3.3 in para-aortic lymph nodes was predictive of worse overall survival in patients with para-aortic lymph node metastasis, while Kidd et al. [25] showed max SUV in the pelvic lymph node was an independent predictor of disease recurrence. As these previous studies [23–25] have shown, baseline lymph node SUV max may have prognostic value; our findings suggest that percent change may not offer additional predictive utility. However, post-treatment lymph node SUV changes likely reflect a complex interplay of treatment response, inflammatory reaction, and reactive changes that may obscure the relationship with persistent disease. Future investigations should incorporate standardized lymph node SUV measurements and correlation with histologic findings when feasible to better elucidate the role of percent change of SUV in the lymph nodes in cervical cancer surveillance.

Recent interest has been given to other markers of disease recurrence on FDG-PET scans. In a retrospective analysis by Pedraza and colleagues [26], they found that cervical max SUV was not an independent prognostic factor in multivariable analysis, while metabolic tumor volume and total lesion glycolysis in combination with texture analysis were significant prognostic factors. Other retrospective and prospective studies have similarly demonstrated that metabolic tumor volume and/or total lesion glycolysis [27, 28] as well as intratumoral heterogeneity [29, 30] are significant prognostic factors in predicting treatment outcome and survival in locally advanced cervical cancer patients. However, these parameters are not routinely collected in many institutions including ours, limiting the clinical application of these findings.

### Local recurrence rates in contemporary context

Our local recurrence rate of 9% (5/55 patients) represents 33% of all recurrences observed in this cohort, which is higher than other reported studies [31–33] warranting comparison against published outcomes with modern radiation techniques. In the EMBRACE-I study, local failure rates ranged from 4 to 8% with modern 3D image-guided brachytherapy targeting EQD2 ≥ 85 Gy to the high-risk clinical target volume [34]. Similarly, recent reports using volumetric dosing demonstrate local control rates of 90–95%.

Our cohort’s local recurrence pattern likely reflects the transition in treatment techniques during our study period. Of the 34 patients treated with point A-based dosing before June 2019, 4 experienced local recurrence (12%), compared to 1 of 21 patients (5%) treated with volumetric 3D-guided brachytherapy after June 2019. While our small numbers preclude statistical comparison, this trend aligns with published literature demonstrating superior local control with modern image-guided techniques that ensure adequate high-risk clinical target volume coverage.

The heterogeneity in brachytherapy techniques within our cohort represents the evolving clinical practice during the study period. This change may have influenced our results and highlights the importance of standardized, image-guided dose delivery when interpreting metabolic response on FDG-PET imaging. Future investigations with uniform modern radiation techniques may provide clearer insights into the relationship between SUV changes and local control.

### Strengths and weaknesses

Strengths of this study include complete data for each patient and pre- and post-treatment SUV. Use of FDG-PET with non-contrast CT has wide applicability across institutions versus a scan with a dedicated diagnostic CT scan that may not be available at all institutions. Limitations include the retrospective nature, single-center, and small sample size, all of which may lead to inherent selection bias. The low number of recurrence events (*n* = 15 total; 5 local, 10 distant) necessitated the use of Firth’s penalized likelihood method for logistic regression analyses, and multivariable models for local recurrence did not converge, limiting our ability to adjust for covariates in these analyses. However, our institution is a tertiary referral and NCI center, which we believe helps to mitigate selection bias risk. The transition from point A-based to volumetric brachytherapy dosing mid-study in 2019 represents a potential confounder, though the consistent EQD2 target of 85 Gy to the tumor provides some standardization. Since the interval from radiation to post-treatment PET scan varied (2–5 months), this variation may have impacted SUV results as well. Use of the date of last clinical follow-up likely underestimated actual overall survival. Progression-free survival data were limited due to the variable start dates of radiation therapy. This led to more longitudinal data in patients who began therapy at the beginning of our designated timeframe (2009) compared to those completing therapy and post-treatment scan just prior to the end of our data-collection timeframe (2021). Lastly, our reliance on radiologist reports of FDG avidity of lymph nodes may have introduced variability, and the inability to distinguish between metabolic changes due to persistent disease versus reactive inflammation without histologic confirmation represents a significant limitation.

### Implications for practice and future research

Further work investigating SUV changes at the primary tumor site in combination with individual patient characteristics as well as individual tumor profiling data may support the development of more accurate, individualized recurrence risk assessments. In addition, further standardization of lymph node assessment with histologic confirmation could clarify the prognostic value of lymph node avidity.

Based on our data, changes in SUV at the primary tumor site and the lymph nodes after treatment may not be reliable stand-alone markers for predicting recurrence or overall or progression-free survival. It may be more useful to combine SUV changes with other patient characteristics and tumor data to predict disease recurrence and guide clinical decision-making.

## Data Availability

No datasets were generated or analyzed during the current study.
